# 2025: The year in which the International Brazilian Journal of Urology will be able to consolidate itself as one of the most important in urology

**DOI:** 10.1590/S1677-5538.IBJU.2025.01.01

**Published:** 2025-01-10

**Authors:** Luciano A. Favorito

**Affiliations:** 1 Universidade do Estado do Rio de Janeiro Unidade de Pesquisa Urogenital Rio de Janeiro RJ Brasil Unidade de Pesquisa Urogenital - Universidade do Estado do Rio de Janeiro - Uerj, Rio de Janeiro, RJ, Brasil; 2 Hospital Federal da Lagoa Serviço de Urologia Rio de Janeiro RJ Brasil Serviço de Urologia, Hospital Federal da Lagoa, Rio de Janeiro, RJ, Brasil

In 2024 there was a small drop in the journal's impact factor, which nevertheless remained above 3. In 2025 we expect the impact factor to reach a very significant level, consolidating our journal as one of the most important in urology. The January-February number of Int Braz J Urol presents original contributions with a lot of interesting papers in different fields: Robotic Surgery, Prostate Cancer, Infertility, Endourology, Priapism, Bladder function, Climacturia and Overactive bladder. The papers came from many different countries such as Brazil, China, Indonesia and USA, and as usual the editor's comment highlights some of them. The editor in chief would like to highlight the following works:

Dr. Tobing and collegues from Indonesia, presented in page e20240356 (1) a nice systematic review comparing the external ureteral catheter and double-J stent as drainage methods for tubeless percutaneous nephrolithotomy (PCNL) and concluded that the external ureteral catheter (EUC) demonstrated fewer stent-related symptoms than the DJ-stent in tube- less PCNL, while both methods showed comparable safety and efficacy. The choice between EUC and DJ-stent should consider patient preferences and surgeon expertise. Further randomized controlled trials (RCTs) with larger sample sizes are needed to affirm these results.

Dr. Ferrão and collegues from Brazil, presented in page e20240406 (2) a nice systematic review about the prevalence of climacturia in patients after radical prostatectomy and concluded that climacturia is a frequently underestimated complication by urologists. Given its significant impact on quality of life, it warrants greater attention from specialists following radical prostatectomy.

Dr. Badia and collegues from the group of Dr. Allen Morey - USA, presented in page e20240497 (3) a important review about the surgical management of ischemic priapism and concluded that the while upfront penile prosthesis placement was previously considered the procedure-of-choice for cases of severe refractory priapism, there has more recently been a paradigm shift towards the utilization of tunneling procedures in the acute setting. The high efficacy of these maneuvers and the potential for sexual function recovery, potentially allow penile prostheses to be avoided in some patients; further studies are needed to investigate this hypothesis. For those ultimately requiring delayed penile prosthesis placement, the increased complication rates should be acknowledged, although multiple techniques exist to facilitate device placement. Ultimately, there is no proven algorithmic approach to the management of this challenging condition; interventional approach remains a nuanced one that depends on both patient and surgeon factors.

Dr. Vieira and collegues from Brazil, presented in page e20240318 (4) a nice study about themorphological (linear measurements) and functional (ADC value) assessments of periprostatic fat can predict the aggressiveness of prostate cancer (PCa) over a 5-year follow-up period. This study is the cover of the present edition. The authors topic concluded that the ADC value of periprostatic fat may serve as an additional tool for PCa risk stratification, correlating with poorer outcomes such as systemic recurrence and overall survival. If validated by external, prospective, multicenter studies, these findings could impact future therapeutic decisions.

Dr. Ferreira and collegues from Brazil, presented in page e20240375. (5) a interesting study about the long-term follow-up of patients undergoing nephrectomy for urolithiasis and concluded that type 2 diabetes mellitus and age were predictors of chronic kidney disease progression, while higher preoperative eGFR was protective. Hypercalciuria and contralateral kidney stones increased the risk of kidney stone formation and/or growth post-nephrectomy for urolithiasis.

Dr. Macedo and collegues from Brazil, presented in page e20240453 (6) a nice study about the management of children and adolescents with overactive bladder refractory to treatment with parasacral transcutaneous electrical nerve stimulation and concluded that children with OAB refractory to pTENS who received structured subsequent treatments showed partial response in all cases, with complete symptom resolution in half of the patients. More intensive urotherapy, medications, or repeat pTENS in combination with oxybutinin can be effective for managing this challenging condition.

Dr. Osório and collegues from Brazil, presented in page e202409922 (7) a interesting research comparing the Gleason 7 (3+4) and (4+3) prostatic adenocarcinomas with prognostic criteria and immunohistochemical profiles of AMCR, PSA and Ki-67 and concluded that differences in the Gleason score 7 (3+4) and Gleason score 7 (4+3) of PC when comparing prognostic criteria. Anti-Ki 67 and anti-PSA antibody immunostaining showed a positive correlation as the Gleason score 7 increased from (3+4) to (4+3).

Dr. Yang and collegues from China, presented in page e20240311 (8) a interesting study about the effect of detethering surgery on the bladder function and psychology of children with primary tethered cord syndrome and concluded that detethering surgery (DS) could not considerably ameliorate pre-existing bladder dysfunction and patients exhibiting non-progressive bladder dysfunction could be treated conservatively with close observation. Tethered cord syndrome (TCS) plagues patients all the time even if detethering. Psychological counseling for children with TCS should be strengthened after DS.

The Editor-in-chief expects everyone to enjoy reading.
